# Optimizing hammer mill speed: Impact on growth performance, pellet quality, and gizzard gene expression in broilers

**DOI:** 10.14202/vetworld.2025.2542-2552

**Published:** 2025-08-30

**Authors:** Taha M. Taha, Mohammad A. Jalal, Hana A. Zakaria, Hosam H. Titi

**Affiliations:** Department of Animal Production, School of Agriculture, The University of Jordan, Amman 11942, Jordan

**Keywords:** broiler chickens, feed conversion efficiency, gizzard morphology, growth performance, hammer mill speed, insulin-like growth factor 2 expression, pellet durability

## Abstract

**Background and Aim:**

Feed constitutes 60%–70% of total poultry production costs, and optimizing feed processing is critical for improving efficiency. This study evaluated the effect of varying hammer mill speeds (HMSs) during corn grinding on growth performance, pellet quality, gizzard morphology, and expression of growth-related genes (growth hormone [GH], GH receptor [GHR], insulin-like growth factors 1 and 2 [IGF1, IGF2]) in broiler chickens.

**Materials and Methods:**

A total of 1,500 one-day-old Ross 308 broiler chicks were randomly divided into three groups: HMS100 (control, 100% HMS), HMS75 (75% HMS), and HMS50 (50% HMS), each with five replicates of 100 birds. Birds were fed isocaloric, isonitrogenous pelleted diets for 28 days. Performance metrics, pellet durability and hardness, gizzard morphology, and intestinal length were assessed. Gene expression analysis in gizzard tissue was conducted using quantita-tive reverse transcription polymerase chain reaction for GH, GHR, IGF1, and IGF2.

**Results:**

Broilers in the HMS75 and HMS50 groups exhibited significantly lower feed intake (by 4.03% and 3.99%) and higher body weight (BW) (by 5.29% and 3.53%) compared to HMS100 (p < 0.05). HMS75 significantly improved feed conversion ratio by 6.81% and BW gain by 5.04% (p < 0.05). Pellet durability and hardness were enhanced in both HMS75 and HMS50 groups (p < 0.05). Gizzard width and muscle thickness were significantly increased at reduced mill speeds, especially in HMS50. Intestinal length was longest in HMS75 (1.96 m). Gene expression analysis revealed a 113% increase in GH expression in HMS75 and a 303% upregulation of IGF2 in HMS50 compared to HMS100 (p < 0.05).

**Conclusion:**

Reducing HMS to 75% optimized broiler growth performance, pellet quality, and intestinal development, while 50% speed promoted IGF2-mediated gizzard hypertrophy. Hammer mill modulation provides a practical strategy to balance feed efficiency and targeted tissue growth in broilers.

## INTRODCTION

Feed expenses constitute approximately 60%–70% of the total cost in poultry production, making feed efficiency a central concern for profitability. To address this challenge, producers must adopt cost-effective nutritional strategies and optimize feed processing techniques [1]. Among these, particle size stands out as a critical factor influencing the nutritive value of poultry diets [2]. Although numerous studies have demonstrated the impact of particle size on animal performance, optimizing feed efficiency remains a persistent challenge [3].

Reducing particle size during grain processing enhances enzyme–digesta interactions, feed consumption, and nutrient absorption, while also improving pellet quality, mixing uniformity, and minimizing post-mixing segregation [4–7]. Corn, a primary energy source in poultry diets, significantly influences pellet quality through its particle size. Notably, corn grinding accounts for nearly 70% of the total energy consumed in feed milling operations [8]. In the United States, corn is typically ground to ~800 μm regardless of the broiler rearing stage [9].

Coarsely ground particles have been shown to improve pellet durability, stimulate reverse peristalsis, and extend digesta retention time in the gastrointestinal tract, thereby enhancing nutrient digestibility in broilers [2, 10–12]. These coarse particles also support digestive tract development and improve feed conversion efficiency by prolonging nutrient exposure to enzymatic digestion [13, 14]. However, while coarser particles promote cohesion during pelleting, they may simultaneously reduce pellet durability and increase the proportion of fines. This underscores the need to balance particle size to achieve optimal pellet quality [15].

On the other hand, finely ground corn improves pellet durability by increasing compaction during pelleting [16]. Hammer mills are favored over roller mills in poultry feed processing due to their lower maintenance demands and higher efficiency [3, 17]. Research by Yousefian Astaneh *et al*. [18] showed that enlarging the hammer mill screen size could reduce corn grinding energy consumption by up to 35%. Furthermore, the integration of variable-frequency drives (VFDs) allows for precise control of tip speed and air flow, thereby enabling more accurate control of particle size distribution [3].

Recent advances in feed manufacturing have been paralleled by genetic selection in broilers, enhancing the expression of molecular growth factors, such as growth hormone (GH) and insulin-like growth factors 1 and 2 (IGF1, IGF2), which are essential for muscle development and performance [19]. Selective breeding has notably improved feed efficiency through elevated GH levels, underscoring the synergistic relationship between nutrition and growth potential [19]. IGF1 and IGF2 are especially important for broiler growth, as they regulate key cellular processes involved in muscle and gizzard development [20]. Moreover, dietary particle size has been shown to significantly influence gizzard morphology, which is tightly linked to growth performance. Notably, IGF2 also plays a role in glucose metabolism by activating its receptor in skeletal muscle tissue [20].

While numerous studies have investigated the effects of feed particle size on broiler growth performance, digestive tract development, and pellet durability, most have focused primarily on the influence of screen size or feed form (mash vs. pellet) without directly examining hammer mill speed (HMS) modulation as a processing parameter. Moreover, previous research has predominantly evaluated performance and morphological outcomes without integrating molecular insights, particularly gene expression linked to growth regulation. To date, no comprehensive studies have simultaneously assessed the impact of HMS on growth performance, pellet characteristics, gizzard morphometry, and gene expression in broilers. Notably, the relationship between corn particle size, modulated through mill speed, and the expression of key growth-regulating genes such as GH, GH receptor (GHR), IGF1, and IGF2 in gizzard tissue remains largely unexplored. This represents a critical gap in our understanding, especially given the role of the GH–IGF axis in coordinating muscular development, nutrient utilization, and organ adaptation in broilers.

This study aimed to evaluate the impact of HMS reduction during corn grinding on broiler chicken growth performance, feed conversion efficiency, pellet durability, and gastrointestinal morphology, with a special focus on the expression of growth-related genes in the gizzard. By investigating three distinct HMSs (100%, 75%, and 50%), the study sought to identify the optimal processing condition that balances physical feed quality with physiological and molecular outcomes. Specifically, the study explored whether reduced milling speeds, leading to coarser particle sizes, could enhance gizzard development and stimulate the expression of GH, GHR, IGF1, and IGF2, thereby improving digestive efficiency and growth performance. This integrative approach bridges feed technology and molecular biology, offering a novel perspective on how feed processing variables can modulate gene-level responses in broilers.

## MATERIALS AND METHODS

### Ethical approval

All animal procedures were conducted in accordance with institutional guidelines for animal care and use. Ethical approval was granted by the Deanship of Scientific Research, University of Jordan (Approval No. 2246/37).

### Study period and location

The study was conducted for 28 days (between 22 January and 23 February 2023) in a climate-controlled poultry research facility located in Al Taffeh, Az Zarqa Governorate, northeast of Amman, Jordan.

### Experimental design

A total of 1,500 one-day-old straight-run Ross 308 broiler chicks were procured from a commercial hatchery (Altahoneh, Al-Zarqa, Jordan). Chicks were randomly allocated in a completely randomized design into three dietary treatment groups as follows:

- HMS100 (Control): 100% HMS (123,419 × *g*)
- HMS75: 75% HMS (69,424 × *g*)
- HMS50: 50% HMS (30,855 × *g*) Each treatment included five replicates of 100 birds (n = 500 birds/treatment group).

### Bird management and housing

Birds were raised under standard husbandry practices in a fully enclosed, environmentally controlled facility. Room temperature was initially maintained at ~32°C and gradually decreased to ~24°C by day 28, following Ross 308 management guidelines (Aviagen, Huntsville, Alabama, USA). Birds were reared in floor pens (1.0 m × 5.0 m) lined with wood shavings, with *ad libitum* access to feed and water. Feed trays were used during the first 5 days, after which cylindrical feeders were introduced for the remainder of the study.

### Diet formulation and nutrient analysis

All treatment groups received isocaloric and isonitrogenous pelleted diets formulated using Bestmix 3.33 software (Bestmix, Maldegem, Belgium), to meet Ross 308 nutritional requirements for the grower and finisher phases. Before formulation, corn was ground using a 4-mm sieve at the specified HMSs. Proximate analysis was performed according to the Association of Official Analytical Chemists (AOAC) official methods: dry matter, ash (AOAC 942.05), and crude protein (AOAC 954.01). Table 1 details ingredient composition and nutrient values.

**Table 1:** Ingredient composition and calculated nutrient values of the starter (1–11 days), grower (12–22 days), and finisher (23–28 days) diets.

Ingredient	Starter grower finisher		

	(1–11 days)	(12–22 days)	(23–28 days)

	(%)		
Corn	57.71	59.80	65.20
Soybean meal (48%)	37.47	35.34	29.84
Soybean oil	1.80	2.60	2.70
Monocalcium phosphate	0.90	0.60	0.60
Limestone	0.14	0.11	0.11
DL-Methionine	0.41	0.325	0.325
L-Lysine	0.375	0.275	0.275
L-Arginine	0.125	0.035	0.035
L-Threonine	0.175	0.10	0.10
L-Valine	0.125	0.045	0.045
L-isoleucine	0.035	0.035	0.035
Sodium chloride	0.19	0.20	0.20
Choline chloride (72%)	0.05	0.04	0.04
Vitamin premix^1^	0.10	0.10	0.10
Trace mineral premix^2^	0.10	0.10	0.10
Ronozyme hiphos^3^	0.01	0.01	0.01
Rovabio advaned P^4^	0.005	0.005	0.005
Ronozyme ProAct^5^	0.015	0.015	0.015
Antioxidant	0.010	0.010	0.010
Calculated nutrient composition			
ME, kcal/kg feed	3030	3110	3180
Crude protein (%)	22.50	21.50	19.40
Ether extract (%)	4.00	4.70	4.87
Ash (%)	5.66	5.00	4.70
Digestible lysine (%)	1.38	1.27	1.13
Digestible methionine (%)	0.74	0.64	0.62
Methionine+Cysteine (%)	1.05	0.95	0.90
Digestible threonine (%)	0.89	0.80	0.87
Digestible arginine (%)	1.48	1.34	1.18
Digestible valine (%)	1.05	0.96	0.87
Ca (%)	1.00	0.82	0.80
Nonphytate phosphorus (%)	0.50	0.43	0.42
Na (%)	0.17	0.17	0.17
K (%)	1.30	1.30	1.22
CI (%)	0.20	0.22	0.22
Linoleic acid (%)	2.60	3.06	3.21

1 Vitamin premix per kilogram of diet: Vitamin A, 13,000 IU; Vitamin D3, 4000 IU; Vitamin E, 100 mg; Vitamin B1, 3 mg; Vitamin B2, 9 mg; Vitamin B6, 6 mg; Vitamin B12, 0.4 mg; folic acid, 2 mg; biotin, 0.25 mg.

2 Trace mineral premix provided per kilogram of diet: 50 mg of iron carbonate, 100 mg of manganese oxide, 12 mg of copper sulfate, 100 mg of zinc, 1.6 mg of calcium iodide, 3 mg of sodium selenite, and 0.4 mg of cobalt sulfate.

3 Ronozyme hiphos: contains a minimum of 20,000 units of Phytase per gram.

4 Rovabio advanced P: contained per gram a minimum of 22,000 units β-xylanase; 2000 units β-glucanase.

5 Ronozyme ProAct=It contains a minimum of 75,000 units per gram of protease, ME=Metabolizable energy

### Hammer mill and pelleting parameters

Corn grinding was performed using an SFSP132 × 50C/A hammer mill (Famsun, Yanghou, China) equipped with a 1,320-mm rotor diameter, 500-mm width, and 64 hammers, operating through a Yaskawa (Fukuoka, Japan) A1000 132 kW VFD. Milling speeds were adjusted to 1,486 rpm (100%), 1,114 rpm (75%), and 743 rpm (50%) by varying the frequency between 50 Hz, 37.5 Hz, and 25 Hz, respectively. Tip speeds were 492 m/s (100%), 369 m/s (75%), and 246 m/s (50%). Milling samples were collected over a 1-h operation.

Pelleting was carried out using a MUZL610 × 170 pellet mill (Famsun) with a 4.0 × 52 mm die hole. Pellet production capacity was monitored using a Siemens (Munich, Germany) digital controller. Conditioner temperature was maintained at 80°C ± 2°C, and mill operation was regulated to 90% of motor load (220 ± 3 amps).

### Performance parameters

Performance metrics included feed intake (FI), body weight (BW), body weight gain (BWG), feed conversion ratio (FCR), and mortality. FI was recorded daily, whereas BW was measured weekly using 10 randomly selected birds per replicate. FCR was calculated as:

FCR = Total FI (g)/Total BWG (g)

Mortality-adjusted FCR and FI were determined by incorporating the weights of deceased birds into the total pen gain.

### Pellet quality assessment

Pellet durability index (PDI) was determined using Ensminger’s method [21]:

PDI (%) = (Weight after tumbling/Weight before tumbling) × 100

Pellet hardness was measured using a Hercules M durometer (Amandus KAHL GmbH & Co. KG, Reinbeck, Germany) on 10 pellets per sample. A load was applied to measure indentation force in kilogram-force (kgf). For each feed treatment, 20 samples were analyzed for PDI and hardness [22].

### Gizzard and intestinal morphometry

On day 28, eight birds per replicate (n = 40/treatment group) were selected for morphometric assessment. Birds were fasted for 8 h, electrically stunned, exsanguinated, scalded (60°C for 45 s), defeathered, and manually eviscerated. The proventriculus, gizzards, and small intestines were excised.

Measurements included:

- Gizzard and proventriculus weights (digital scale, BAT1, 1 g precision)
- Gizzard length and width
- Thickness of upper, middle, and lower muscle layers (digital caliper)
- Intestinal length (measuring tape).

Methods followed established anatomical protocols [23–26].

### Gene expression analysis by quantitative reverse transcription polymerase chain reaction (qRT-PCR)

#### Sample collection and RNA extraction

Gizzard muscle samples from each region (upper, middle, lower) were pooled from 8 birds per replicate (n = 40/treatment) and preserved in Trizol. Homogenization was performed using 1.8-mm ceramic beads in Omni tubes with the Omni Bead Ruptor 4 (Omni International, USA). RNA was extracted using the automated nucleic acid extraction system to isolate nucleic acid (XT PGS) kit on the Miracle Automated Extraction System (iNtRON Biotechnology, South Korea), and purity was verified using a NABI spectrophotometer (MicroDigital, Korea) [27].

#### Complementary DNA (cDNA) synthesis and quantitative polymerase chain reaction protocol

cDNA synthesis was conducted using PrimeScript Reverse Transcriptase Master Mix (Takara, Japan). Gene expression was quantified using TB Green Premix Ex Taq II (Takara) on a Quant Gene 9600 (BIOER Technology, Hangzhou, China). Reaction volume: 20 μL (2 μL cDNA, 10 μL mix, 1 μL primers, 6 μL nuclease-free water) [28].

#### Thermal cycling conditions

- Initial denaturation: 95°C for 30 s
- 40 cycles: 95°C for 15 s, 60°C for 30 s
- Melting curve: 60°C–95°C in 0.3°C increments.

#### Normalization and validation

Glyceraldehyde-3-phosphate dehydrogenase (GAPDH) was used as the reference gene. Primer specificity was verified using melting curve analysis and National Center for Biotechnology Information-The Basic Local Alignment Search Tool. Amplification efficiencies were: GAPDH (100.25%), IGF1 (102.49%), and IGF2 (103.91%).

### Primer design

Primers were adopted from published sources: GH and GHR from Engberg *et al*. [29]; IGF1 and IGF2 from Saxena *et al*. [30]. Table 2 summarizes primer sequences, annealing temperatures, and amplicon sizes. Primer specificity and efficiency were validated using standard curves generated from serial cDNA dilutions.

**Table 2:** Primer sequences used in real-time qPCR analysis.

Gene	Sequence	Anneal. temp*	Amplicon size (bp)	NCBI reference sequence
*Gallus gallus* (GH)	*F: 5′ CACCACAGCTAGAGACCCACATC 3′ *R: 5′ CCCACCGGCTCAAACTGC 3′	63	201	NM_205518.2
*Gallus gallus* (GHR)	F: AACACAGATACCCAACAGCC R: AGAAGTCAGTGTTTGTCAGGG	63	291	NM_204524.2
*Gallus gallus* IGF1 expression	F: CACCTAAATCTGCACGCT R: CTTGTGGATGGCATGATCT	63	146	NM_204628.2
*Gallus gallus* IGF2 levels	F: TCTTCCCGTAACCACGTCCC R: ATTGCTGAGGCAGTCATGCG	63	131	NM_001004414.4

* Annealing temperature. GH = Growth hormone, GHR = Growth hormone receptor, IGF = Insulin-like growth factor 1, IGF2 = Insulin-like growth factor 2, bp = Base pairs, NCBI = National Center for Biotechnology Information, qPCR = Quantitative polymerase chain reaction, *F: forward, *R: Reverse

#### Statistical analysis

Data were analyzed using Statistical Analysis System (SAS) software (version 9.4; SAS Institute, Cary, NC, USA) [31] with the PROC MIXED procedure. Shapiro–Wilk and Levene’s tests were used to assess normality and homogeneity of variance. One-way analysis of variance followed by Tukey’s *post hoc* test was applied for mean comparisons (p < 0.05). The general linear model used was:

Y_ij_ = μ + τ_i_ + ε_ij_

Where:

- Y_ij_ = observation of the j^th^ replicate in the i_th_ treatment
- μ = overall mean
- τ_i_ = fixed effect of treatment
- ε_ij_ = random error assumed to follow N(0, σ^2^).

## RESULTS

### Growth performance

Cumulative performance outcomes across the 28-day trial are presented in Table 3. FI was significantly higher in the HMS100 (control) group compared to HMS75 and HMS50, with reductions of 4.03% and 3.99%, respectively (p < 0.05). At day 28, birds in the HMS75 and HMS50 groups had significantly higher BW than those in the HMS100 group by 5.29% (90 g) and 3.53% (60 g), respectively (p < 0.05).

**Table 3:** Effect of particle size on cumulative feed intake, body weight, feed conversion ratio, body weight gain, and mortality rate.

Performance parameters	Age (Weeks)	Treatments^1^				

		HMS100	HMS75	HMS50	SEM^2^	p-value
FI^3^ (g/bird)	1	160.40	155.20	158.20	2.64	NS
	2	540.60	539.60	544.20	7.99	NS
	3	1251.60	1264.80	1267.60	21.12	NS
	4	2263.40^a^	2172.2^b^	2173.2^b^	37.00	0.05
BW^4^ (g)	1	200	200	210	0.0022	NS
	2	540	550	550	0.0062	NS
	3	1100	1150	1150	0.0193	NS
	4	1700^b^	1790^a^	1760^a^	0.0175	0.01
FCR^5^ (g: g)	1	0.79	0.76	0.77	0.0174	NS
	2	1.01	0.98	0.99	0.0187	NS
	3	1.13	1.10	1.11	0.0245	NS
	4	1.32^b^	1.23^a^	1.27^ab^	0.0280	0.04
BWG^6^ (g)	1	160	160	160	0.0022	NS
	2	490	500	500	0.0075	NS
	3	900	940	940	0.0206	NS
	4	1190^b^	1250^a^	1200^b^	0.0213	0.01
Mortality (%)	1	0.05	0.03	0.03	0.0088	NS
	2	0.07	0.06	0.07	0.0090	NS
	3	0.09	0.09	0.09	0.0125	NS
	4	0.10	0.11	0.09	0.0160	NS

^a,b^Means within rows with varying superscripts differ significantly (p < 0.05).

1 Dietary treatments: HMS100 = Hammer mill speed (100%), HMS75 = Hammer mill speed (75%), and HMS50 = Hammer mill speed (50%). The sieve size was 4 mm for all treatments,

2 SEM = Standard error of the mean, FI

3 = Feed intake, BW

4 = Body weight, FCR

5 = Feed conversion ratio, BWG

6 = Body weight gain, NS = Non-significant

FCR was significantly improved in the HMS75 group, showing a 6.81% enhancement over HMS100 (p < 0.05). However, FCR values did not differ significantly between HMS75 and HMS50. BWG was also significantly greater in HMS75 compared to both HMS100 and HMS50 (p < 0.05). Mortality rates did not significantly differ among the three treatment groups.

### Pellet quality

Pellet characteristics are summarized in Table 4. Both the PDI and hardness increased significantly with decreasing HMSs. The HMS75 and HMS50 groups showed PDI values of 93.90% and 94.26%, representing 1.0% and 1.4% improvements over HMS100 (p < 0.05), respectively.

**Table 4:** Pellet durability index and hardness.

Pellet quality tests	Treatments^1^				
	HMS100	HMS75	HMS50	SEM^2^	p-value
PDI^3^	92.97^b^	93.90^a^	94.26^a^	1.35	0.004
Hardness	2.20^b^	2.90^a^	3.00^a^	0.49	0.001

^a,b^Means within rows with varying superscripts differ significantly (p < 0.05).

1 Dietary treatments: HMS100 = Hammer mill speed (100%), HMS75 = Hammer mill speed (75%), and HMS50 = Hammer mill speed (50%). The sieve size was 4 mm for all treatments,

2 SEM = Standard error of the mean, PDI

3 = Pellet durability index

Pellet hardness followed a similar trend, increasing by 31.8% in HMS75 (2.90 kgf) and 36.4% in HMS50 (3.00 kgf), compared to HMS100 (2.20 kgf) (p < 0.05).

### Gizzard and intestinal morphology

Morphometric data are detailed in Table 5. Gizzard width was significantly greater in the HMS75 group (75.08 mm), showing increases of 5.61% and 6.57% over HMS100 (71.09 mm) and HMS50 (70.45 mm), respectively (p < 0.05). The middle gizzard muscle layer was significantly thicker in HMS50 (12.87 mm) than in HMS75 (11.37 mm) and HMS100 (9.86 mm), with increases of 13.19% and 30.53%, respectively (p < 0.05).

**Table 5:** Mean gizzard and proventriculus weights, gizzard length and width, gizzard muscle thickness, and intestine length.

Intestinal parameter	Treatments^1^				

	HMS100	HMS75	HMS50	SEM^2^	p-value
Gizzard weight (g)	22.41	24.69	24.53	2.64	NS
Proventriculus weight (g)	7.191	7.87	7.97	1.01	NS
Length of the gizzard (mm)	46.391	45.10	47.69	4.21	NS
Gizzard Width (mm)	71.09^b^	75.08^a^	70.45^b^	3.16	0.01
Gizzard muscle thickness (mm)					
Upper region	1.51	1.57	1.52	0.32	NS
Middle region	9.86^c^	11.37^b^	12.87^a^	1.66	0.001
Lower region	0.62^b^	0.75^b^	1.23^a^	0.44	0.01
Length of the small intestine (m)	1.88^ab^	1.96^a^	1.72^b^	0.21	0.05

^a,b,c^Means within rows with varying superscripts differ significantly (p < 0.05).

1 Dietary treatments: HMS100 = Hammer mill speed (100%), HMS75 = Hammer mill speed (75%), HMS50 = Hammer mill speed (50%). The sieve size was 4 mm for all treatments.

2 SEM = Standard error of the mean, NS = Non-significant

Similarly, the lower gizzard muscle region was significantly thicker in HMS50 (1.23 mm), increasing by 98.39% over HMS100 (0.62 mm) and 64.00% over HMS75 (0.75 mm) (p < 0.05). Intestinal length was longest in the HMS75 group (1.96 m), 13.95% greater than HMS50 (1.72 m) (p < 0.05). No significant differences were observed in gizzard or proventriculus weights, gizzard length, or upper muscle layer thickness across the groups.

### Gene expression profiles

Gene expression results are shown in Table 6. Birds in the HMS50 group exhibited significantly elevated levels of chicken GH (CGH) and IGF2 in gizzard tissue compared to HMS100 and HMS75 (p < 0.05). CGH levels increased by 113% (2.77 vs. 1.30), and IGF2 expression rose by 303% (6.94 vs. 1.72) relative to the control. These changes correspond to the enhanced gizzard muscularity observed in HMS50.

**Table 6:** qRT-PCR results for GHR, CGH, IGF1, and IGF2.

Variables	Treatments^1^				

	HMS100	HMS75	HMS50	SEM^2^	p-value
GHR^3^	1.16	1.60	1.56	0.58	NS
CGH^4^	1.30^b^	2.77^b^	6.24^a^	64.23	0.02
IGF1^5^	1.18	1.06	1.06	0.047	NS
IGF2^6^	1.72^b^	2.20^b^	6.94^a^	83.14	0.04

^a, b^Means within rows with varying superscripts differ significantly (p < 0.05).

1 Dietary treatments: HMS100 = Hammer mill speed (100%), HMS75 = Hammer mill speed (75%), HMS50 = Hammer mill speed (50%), and sieve size of 4 mm for all treatments.

2 SEM = Standard error of the mean, GHR

3 = Growth hormone receptor, CGH

4 = Chicken growth hormone, IGF1

5 = Insulin-like growth factor 1, IGF2

6 = Insulin-like growth factor 2, NS = Non-significant

No statistically significant differences were observed in the expression of GHR or IGF1 among the three groups.

## DISCUSSION

### Growth performance and FI

The present findings showed a reduction in FI accompanied by an improvement in FCR. The reduced FI, along with improved growth, may be attributed to enhanced nutrient use in broilers receiving diets processed at 75% and 50% HMSs. Chewning *et al*. [6] reported higher intake with coarse mash diets, implying that pellet hardness (Table 4) may transiently suppress consumption until birds adapt to the physical feed structure. Variations in FI may also be explained by changes in the physical characteristics of the feed that led to reduced consumption but improved nutrient absorption. The enhanced FCR reflects the adaptive capacity of broilers to efficiently convert feed into body mass, underscoring the significance of feed processing parameters in optimizing growth performance [32, 33].

These results contrast with previous reports by Ebbing *et al*. [7], Pacheco *et al*. [10], and Iskakov *et al*. [15], indicating that larger particle size increases FI but reduces feed efficiency and that the physical form of the feed complicates the interpretation of the effects of particle size on nutrient utilization. The increased BW by 4.0% and 6.8% improved FCR compared to full-speed milling (p < 0.05; Table 3), observed in the HMS75% group, can be attributed to the feed particle sizes produced at this speed, which appear to be best for nutrient absorption. Consistent with our results, Zhao *et al*. [34] found that coarse particle sizes facilitated higher digestibility rates and improved overall feed utilization efficiency in broilers.

Moreover, these findings are consistent with those reported by Amerah *et al*. [35], who suggested that appropriate feed particle size fosters improved nutrient availability, which is pivotal for bolstering poultry growth performance. Thus, the increased BWG can be directly linked to the enhanced digestion efficiency of the optimal feed particle size at HMS75. Previous studies by Svihus *et al*. [12] and Zhao *et al*. [34] have concluded that an appropriate particle size enhances nutrient bioavailability, leading to better growth performance. Furthermore, Amerah *et al*. [35] corroborated the ability of lower milling speeds to improve nutrient availability, emphasizing that optimally sized feed particles can significantly improve overall growth.

### Pellet quality and durability

The PDI and hardness are key indicators of feed quality and provide critical insight into the physical characteristics of pellets. Although the experimental diets produced larger particle-size pellets, both PDI and hardness values remained within the acceptable range outlined in the commercial strain guidelines of Ross 308. Improved pellet durability is essential for reducing feed wastage, maintaining pellet integrity, and enhancing production efficiency. Pacheco *et al*. [10] reported that PDI tends to decrease with increasing particle size, although improved feed formulation can mitigate this effect and enhance pellet durability.

In contrast, our results showed that larger corn particles from reduced milling speeds yielded higher PDI and hardness than the control group. This increased pellet hardness may have contributed to the lower FI observed in the HMS75 and HMS50 groups (Table 3) [33]. Rueda *et al*. [33] similarly observed higher PDI values in diets containing larger than finer corn particle sizes. Singh *et al*. [36] also reported a positive linear relationship between whole corn inclusion levels (0%–60%) and pellet durability in broiler diets.

Greater pellet hardness enhances physical pellet integrity and promotes gizzard development, thereby improving the digestive tract’s mechanical feed processing [36]. These findings suggest that achieving a balance between grinding for nutrient digestibility and maintaining adequate pellet quality is essential for optimal broiler feed utilization. Lower HMSs resulted in larger feed particle sizes, revealing a direct relationship between reduced milling speed, decreased FI, and increased weight gain in broilers [33].

The reduced FI in the HMS75 and HMS50 groups may also be explained by the higher pellet durability and hardness, making the feed less palatable [33]. Jha and Das [32] confirmed that feed texture plays a crucial role in feeding behavior and intake, reinforcing the importance of optimizing milling conditions to enhance growth outcomes.

### Gizzard development and intestinal morphometry

The gizzard is a highly responsive organ that adjusts in size and muscularity according to dietary stimulation. Numerous studies have shown that dietary particle size can rapidly influence gizzard development by increasing its volume, muscularity, or both [12]. Amerah *et al*. [35] noted that the wall mass-to-content ratio influences the shearing efficiency of the gizzard, which depends on the physical properties of the diet. These findings imply that moderate milling (75% speed) may optimize gizzard function better than coarse (50%) or fine (100%) grinding.

**Figure 1:**
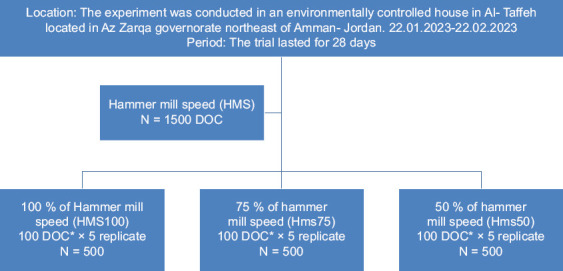
Flow diagram showing the experimental design of the study. *DOC = One-day-old chicks.

Well-developed gizzards with stronger musculature are more effective at grinding feed, particularly in birds that consume pelleted diets [37]. Increased gizzard muscle thickness supports greater grinding force, thereby improving mechanical digestion [12]. When the gizzard volume disproportionately expands to the wall mass, its shearing efficiency declines, reducing the likelihood of particle breakdown during transit [34, 38].

Coarser feed particles extend digesta retention in the gizzard, thereby promoting muscular stimulation and enzymatic digestion, ultimately increasing the weight of the gizzard and related digestive organs [12, 38, 39]. In addition, coarse feed must be reduced to a critical particle size before it can exit the gizzard [12]. A similar delay in digesta transit has been observed in birds fed a whole wheat diet [40].

The observed link between longer intestinal length and better nutrient absorption highlights the need to optimize feed structure for maximal digestive efficiency [41, 42]. Previous studies by Nir *et al*. [40] have reported greater gizzard and ileum weights in birds fed pelleted diets, supporting our findings of enhanced gastrointestinal development with coarser feed. Frikha *et al*. [41] similarly observed that by day 45, corn-fed pullets had heavier digestive tracts, enlarged proventriculi, and longer intestines.

Taylor and Jones [43] reported increased duodenal length in birds fed pelleted diets containing 200 g/kg whole wheat. Gabriel *et al*. [44] observed a 16% reduction in jejunal length in broilers fed with whole wheat, which is consistent with the pattern of intestinal adaptation to feed particle size. These results contrast with those of Amerah *et al*. [35], who noted shorter small intestines in 21-day-old broilers fed coarser corn. Singh *et al*. [36] reported morphological improvements in the upper intestine of whole wheat-fed broilers, potentially enhancing nutrient uptake.

Svihus *et al*. [38] reported that fast digesta transit reduces absorption time, whereas slower transit can limit intake capacity. These patterns are consistent with our findings, where moderate particle size from 75% milling speed led to increased intestinal length and improved FCR.

### Gene expression and growth molecular indicators

To the best of our knowledge, limited data exist on gene expression in gizzard tissue in relation to feed particle size and HMS, making this study a novel contribution to the field. This gap in the literature underscores the novelty and relevance of our findings. Increased IGF2 expression plays a vital role in promoting muscle development and reflects the coordinated GH–IGF signaling activity required for optimal poultry growth [20].

Although IGF1 levels did not differ significantly in this study, prior research by Al-Hassani *et al*. [20] indicates that IGF1 has a synergistic role with GH in supporting growth. Karabag *et al*. [45] demonstrated that dietary modifications can influence the expression of IGF1, thereby affecting muscle development and general health. The increased BW and gizzard development in the HMS50 group may be partially attributed to the elevated IGF2 expression levels.

Al-Hassani *et al*. [20] confirmed that feed processing affects GH and IGF availability, noting that excessively large particles may impair hormonal activation. These findings are consistent with our results, suggesting that optimized milling–particularly reduced HMS–can improve both nutrient profiles and digestive tract function.

Although not statistically significant, the elevated GHR expression observed in HMS50 supports the importance of effective receptor signaling for GH responsiveness in broilers [46]. Novotný *et al*. [46] reported that growth receptor expression strongly correlates with performance outcomes, indicating the capacity of the organism to respond to growth-promoting hormones. Zhao *et al*. [34] emphasized that environmental inputs, particularly nutrition, play a decisive role in determining growth rate, body size, and final weight.

**Figure 2:**
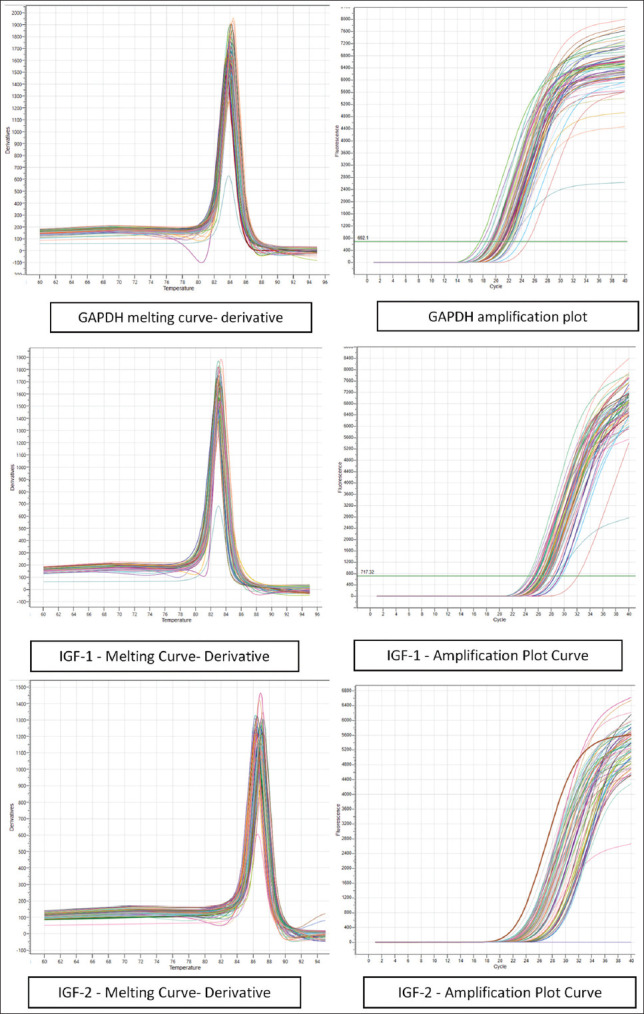
Amplification and melting curves of insulin-like growth factor-1 and insulin-like growth factor-1.

The anabolic effects of GH are largely mediated through circulating or locally synthesized IGF1, a key regulator of myogenesis and muscle mass development [47]. Coarser particles (HMS50) increase gizzard workload, triggering muscular hypertrophy (Table 5), and mechanosensitive signaling. Stretch-activated receptors (e.g., integrins, Piezo channels) in the gizzard wall may stimulate GH release from the pituitary through vagal afferents, explaining the 113% higher CGH levels in HMS75 versus HMS100.

This is consistent with previous studies by Al-Hassani *et al*. [20] and Vaccaro *et al*. [47] showing that the physical feed structure modulates GH secretion, independent of nutrient composition. The observed 303% surge in IGF2 expression in HMS50 (Table 6) is likely attributable to prolonged NR. Coarser particles slow digestion, enhancing the exposure of intestinal nutrient sensors to amino acids and glucose, which are key stimulants of IGF2 [20].

Local production of gizzard smooth muscle expresses IGF2, which is upregulated by mechanical stress to support tissue growth [47]. In contrast, IGF1 remained unchanged, suggesting that particle size selectively activated IGF2’s role in muscle hyperplasia (vs. IGF1’s metabolic functions) [30]. Gizzard hypertrophy in HMS50 (Table 5) may amplify the autocrine effects of IGF2, creating a feed-forward loop:

Mechanical stress → IGF2 → muscle growth → increased grinding capacity → further IGF2 upregulation

This explains why HMS75 (moderate particles) optimized growth performance, whereas HMS50’s extreme coarseness prioritized gizzard adaptation over efficiency. HMS75’s balance: moderately coarse particles (75% speed) maximized nutrient absorption without overstimulating IGF2, avoiding energy diversion to gizzard hypertrophy. HMS50’s niche: muscle accretion is prioritized over feed efficiency in late-stage broilers.

## CONCLUSION

This study demonstrates that modifying HMS significantly influences feed particle size, which in turn affects broiler performance, pellet quality, gastrointestinal development, and growth-related gene expression. Specifically, reducing HMS to 75% (HMS75) improved BW by 4.0%, enhanced FCR by 6.8%, and increased intestinal length and gizzard width, thereby optimizing nutrient digestibility without compromising pellet durability. In contrast, extremely coarse grinding at 50% speed (HMS50) triggered greater gizzard muscle development and significantly upregulated IGF2 (303%) and CGH (113%) expression, highlighting the physiological adaptation to mechanical workload in the digestive tract.

The findings underscore the feasibility of using moderate milling speeds (75%) as a cost-effective strategy to enhance broiler growth performance, reduce energy costs in feed manufacturing, and promote gut health. These adjustments can be readily implemented in commercial feed mills without requiring additional infrastructure, offering an economical means to improve feed efficiency and production sustainability.

This is the first study to evaluate GH and IGF gene expression specifically in gizzard tissue in response to HMS variations. Comprehensive assessment of molecular, morphological, and performance parameters allows for a multidimensional understanding of dietary particle size effects. The use of VFD-controlled milling provides a practical model for industrial feed processing optimization.

However, the study was conducted over a 28-day period, which may not fully capture long-term effects on carcass traits or metabolic health. The mechanistic pathways of GH–IGF signaling activation were inferred but not directly validated through histological or receptor binding studies. The research was limited to a single broiler strain (Ross 308), and results may vary with genotype or environmental conditions.

Future studies should explore longitudinal effects on muscle fiber characteristics, carcass yield, and immune competence to assess whole-bird productivity. Investigating additional gene markers and endocrine pathways involved in nutrient sensing and stress responses could deepen mechanistic insights. Evaluations across different feed compositions, broiler strains, and age phases would help generalize these findings for broader application.

In summary, optimizing HMS emerges as a pivotal lever in feed processing that can balance economic efficiency with biological performance. A moderate reduction to 75% of full mill speed offers the most favorable trade-off between digestive organ stimulation and feed conversion efficiency, avoiding the excessive gizzard hypertrophy associated with overly coarse feed. These findings contribute meaningful insights for poultry nutritionists, feed technologists, and integrators aiming to enhance production outcomes through precision feed engineering.

## AUTHORS’ CONTRIBUTIONS

TMT: Conducted field experiments and manufacturing, sampling, laboratory analysis, and drafted the manuscript. MAJ: Experimental design, data organization, and statistical analysis. HAZ: Sample collection and analysis, diet formulation, and drafted the manuscript. HHT: Experimental design, supervised laboratory analysis, and edited the manuscript. All authors have read and approved the final manuscript.

## References

[1] Donohue M, Cunningham D (2009). Effects of grain and oilseed prices on the costs of US poultry production. J. Appl. Poultry Res.

[2] Saensukjaroenphon M, Evans C.E, Jones C.K, Stark C.R, Paulk C.B (2022). Effect of feed form, corn particle size, and extrusion of corn on broiler performance. KAES Res. Rep.

[3] Braun M, Wecker H, Dunmire K, Evans C, Sodak M.W, Kapetanovich M, Shepherd J, Fisher R, Coble K, Stark C, Paulk C (2021). Evaluation of hammermill tip speed, air assist, and screen hole diameter on ground corn characteristics. Processes.

[4] Berk J (2013). Quality assurance and control in feed manufacturing. J. Anim. Nutr.

[5] Wolfrum S, Siegert W, Rubio-Cervantes I, Feuerstein D, Camarinha-Silva A, Rodehutscord M (2025). Effects of feed particle size, calcium concentration and phytase supplementation on InsP6 degradation in broiler chickens fed pelleted diets. Br. Poult. Sci.

[6] Chewning C.G, Stark C.R, Brake J (2012). Effects of particle size and feed form on broiler performance. J. Appl. Poult. Res.

[7] Ebbing M.A, Yacoubi N, Naranjo V, Sitzmann W, Schedle K, Gierus M (2022). Towards large particle size in compound feed:Using expander conditioning prior to pelleting improves pellet quality and growth performance of broilers. Animals.

[8] Dabbour M.I, Bahnasawy A.B, Ali S, El-Haddad Z (2015). Grinding parameters and their effects on the quality of corn for feed processing. J. Food Process. Technol.

[9] Rubio A.A, Hess J.B, Berry W.D, Dozier W.A, Pacheco W.J (2020). Effects of corn particle size on broiler performance during the starter, grower, and finisher periods. J. Appl. Poult. Res.

[10] Pacheco W.J, Stark C.R, Ferket P.R, Brake J (2013). Evaluation of soybean meal source and particle size on broiler performance, nutrient digestibility, and gizzard development. Poult. Sci.

[11] Péron A, Bastianelli D.F, Oury X, Gomez J, Carre B (2005). Effects of food deprivation and particle size of ground wheat on digestibility of food components in broilers fed on a pelleted diet. Br. Poult. Sci.

[12] Svihus B, Juvik E, Hetland H, Krogdahl A (2004). Causes for improvement in the nutritive value of broiler chicken diets with whole wheat instead of ground wheat. Br. Poult. Sci.

[13] Boussaada T, Derradji O, Lakhdari K, Salha Amira B (2022). Effect of particle size of starter diet on broiler chicken performance. Agric. Sci. Digest. A Res. J.

[14] Ghasemi-Aghgonbad A, Olyayee M, Janmohammadi H, Abdollahi M.R, Kianfar R (2024). The Interactive impacts of corn particle size and conditioning temperature on performance, carcass traits, and intestinal morphology of broiler chickens. Animals.

[15] Iskakov R, Gulyarenko A, Bembenek M, Kassym R (2025). Technologies for efficient grinding of plant and animal waste:A review. EUREKA. Phys. Eng.

[16] Wang-Li L, Xu Y, Stark C, Ferket P, Brake J (2020). Effect of dietary coarse corn inclusion on broiler live performance, litter characteristics, and ammonia emission. Poult. Sci.

[17] Downs K.M, Gulizia J.P, Harder G.R, Stafford E.K, Sasia S.J, Pacheco W.J (2023). Corn particle size variation effects on broiler performance, organ weights, and nutrient digestibility during the early growout period (day 1 to 21). J. Appl. Poult. Res.

[18] Yousefian Astaneh I, Chamani M, Mousavi S.N, Sadeghi A.A, Aminafshar M (2023). Effects of feed form (pellet or mash), corn particle size, and Bacillus-based probiotic supplementation on performance traits and digestive tract health of broiler chickens. S. Afr. J. Anim. Sci.

[19] Al-Anbari E, Al-Rekabi M, Attallah O (2023). Study some genetic variance and breeding value on productive performance for local chicken differ in polymorphism of growth hormone gene. IOP Conf. Ser. Earth Environ. Sci.

[20] Al-Hassani A.S, Al-Hassani D.H, Abdul-Hassan I.A (2022). *Insulin-like growth factor* (*IGF-2*) gene polymorphisms influence certain biochemical parameters in broiler chickens. Iraq. J. Agric. Sci.

[21] Ensminger M.E (2023). Processing effects. In:Feed Manufacturing Technology III. Vol. 66. American Feed Industry Association (AFIA), United States.

[22] Nova Etica (2024). 298 DGP Hardness Tester, Ethik Technology.

[23] Berenfeld B.M (2011). Macroscopic, Microscopic and Morphometric Comparative Study of the Gastrointestinal Tract of Commercial B.U.T. Big 6 Turkeys and wild Turkeys. [Dissertation]. Freie Universität Berlin, Germany.

[24] Alshamy Z, Richardson K.C, Hünigen H, Hafez H.M, Plendl J, AlMasri S (2018). Comparison of the gastrointestinal tract of a dual-purpose to a broiler chicken line:A qualitative and quantitative macroscopic and microscopic study. PLoS One.

[25] De Verdal H, Mignon-Grasteau S, Jeulin C, Le Bihan-Duval E, Leconte M, Mallet S, Martin C, Narcy A (2010). Digestive tract measurements and histological adaptation in broiler lines divergently selected for digestive efficiency. Poult. Sci.

[26] Neuhaus N, Lierz M, Möller Palau-Ribes F (2024). Comparative study of gastrointestinal tract size in three parent breeds for the production of dual-purpose organic chickens. Anat. Histol. Embryol.

[27] Bustin S.A, Benes V, Garson J, Hellemans J, Huggett J, Kubista M, Mueller R, Nolan T, Pfaffl M.W, Shipley G.L, Vandesompele J, Wittwer C.T (2009). The MIQE guidelines:Minimum information for publication of quantitative real-time PCR experiments. Clin. Chem.

[28] Livak K.J, Schmittgen T.D (2001). Analysis of relative gene expression data using real-time quantitative PCR and the 2(-Delta Delta C(T)) method. Methods.

[29] Engberg R.M, Hedemann M.S, Jensen B.B (2002). The influence of grinding and pelleting of feed on the microbial composition and activity in the digestive tract of broiler chickens. Br. Poult. Sci.

[30] Saxena R, Saxena V.K, Tripathi N.A, Dev K, Jubeda B, Agarwal R, Goel A (2020). Dynamics of gene expression of hormones involved in the growth of broiler chickens in response to dietary protein and energy changes. Gen. Comp. Endocrinol.

[31] SAS Institute (2023). SAS Statistical Software.

[32] Jha R, Das R (2018). The efficacy of different feed ingredients on broiler performance:A review. J. Anim. Sci. Biotechnol.

[33] Rueda M.S, Bonilla S, de Souza C, Starkey J.D, Starkey C.W, Mejia L, Pacheco W.J (2024). Evaluation of particle size and feed form on performance, carcass characteristics, nutrient digestibility, and gastrointestinal tract development of broilers at 39 d of age. Poult. Sci.

[34] Zhao X, Hu Y, Wang J (2016). The effect of feed particle size on broiler performance, nutrient utilization, and gastrointestinal characteristics. Asian Aust. J. Anim. Sci.

[35] Amerah A.M, Wilcock P, Ravindran V (2007). Influence of feed particle size and feed form on the performance, energy utilization, digestive tract development, and digesta parameters of broiler starters. Anim. Feed. Sci. Technol.

[36] Singh Y, Amerah A.M, Ravindran V (2014). Whole grain feeding:Methodologies and effects on performance, digestive tract development and nutrient utilization of poultry. Anim. Feed. Sci. Technol.

[37] Abdollahi M, Ravindran V, Svihus B (2013). Pelleting of broiler diets:An overview with emphasis on pellet quality and nutritional value. Anim. Feed. Sci. Technol.

[38] Svihus B, Storkås C.G, Neteland M.K, Reierstad S.E, Dhakal S, Hetland H (2024). Effect of grinding and pellet dimensions on performance, digestive tract functionality and feeding behavior of broiler chickens fed diets based on wheat and maize. J. Appl. Poult. Res.

[39] Svihus B (2010). The gizzard:Function, influence of diet structure, and effects on nutrient availability. Worlds Poult. Sci. J.

[40] Nir I, Hillel R, Ptichi I, Shefet G (1995). Effect of particle size on performance. III. Grinding-pelleting interactions. Poult. Sci.

[41] Frikha M, Safaa H.M, Serrano M.P, Jiménez-Moreno E, Lázaro R, Mateos G.G (2011). Influence of the main cereal in the diet and particle size of the cereal on productive performance and digestive traits of brown-egg laying pullets. Anim. Feed. Sci. Technol.

[42] Ravindran V, Abdollahi M.R (2021). Nutrition and digestive physiology of the broiler chick:State of the art and outlook. Animals (Basel).

[43] Taylor R.D, Jones G.P.D (2004). The incorporation of whole grain inclusion in pelleted broiler chicken diets II. Gastrointestinal and digesta characteristics. Br. Poult. Sci.

[44] Gabriel I, Mallet S, Leconte M, Travel A, Lalles J.P (2008). Effects of whole wheat feeding on the development of the digestive tract of broiler chickens. Anim. Feed. Sci. Technol.

[45] Karabag K, Yıldız B, Ersal M (2019). Insulin-like Growth Factor-1 (IGF-1). In:Conference:International Biological, Agricultural and Life Science Congress.

[46] Novotný J, Horáková L, Řiháček M, Zálešáková D, Šťastník O, Mrkvicová E, Kumbár V, Pavlata L (2023). Effect of different feed particle size on gastrointestinal tract morphology, Ileal Digesta viscosity, and blood biochemical parameters as markers of health status in broiler chickens. Animals.

[47] Vaccaro L.A, Porter T.E, Ellestad L.E (2022). The Effect of commercial genetic selection on somatotropic gene expression in broilers:A Potential role for insulin-like growth factor binding proteins in regulating broiler growth and body composition. Front. Physiol.

